# One-day oral polyethylene glycol based cleanout is effective for pre-colonoscopy preparation in children

**DOI:** 10.1186/s12876-018-0895-7

**Published:** 2018-11-07

**Authors:** Ashwath S. Kumar, Brooke L. Beutler, Thomas M. Attard

**Affiliations:** 1 University of Missouri-Kansas City School of Medicine, Kansas City, MO USA; 2 Children’s Mercy Hospital, Kansas City, USA

**Keywords:** Pediatric, Colonoscopy, Preparation, Cleanout, Quality, Polyethylene glycol

## Abstract

**Background:**

The adequacy of pre-procedure preparation is the principal determinant of the quality of colonoscopy in pediatric as in adult patients. There is a lack of consensus, among providers on a standard pre-procedure regimen. Professional society guidelines include the use of Polyethylene glycol (PEG). Herein we report on the provider-assessed adequacy of a one day, age-categorized dosing, PEG based cleanout regimen in children undergoing colonoscopy in a tertiary institution.

**Methods:**

The standard bowel preparation regime at our institution includes an age dependent minimum PEG dosing regimen in addition to clear liquids the day prior to the procedure. We retrospectively abstracted relevant indices including patient demographics, prep quality, procedure impairment, duration and completion from an institutional quality monitoring survey tool between 2015 and 2016 and similarly abstracted prospectively recorded indices that included the dataset above as well as additional fields for procedure deviations and additional laxative use.

**Results:**

A total of 642 procedures (mean age 12.2 years; F: 380) were accrued, nonadherence to the cleanout regimen (7.3%) and additional laxative use (3.1%) were observed in a small proportion of the prospective dataset subjects, adequate cleanout defined as thin or thick liquid but no solids present was reported in 79.5% and 15.8% of cases and impaired study from inadequate cleanout was reported in 11.8% of studies albeit the cecum was reached and the terminal ileum was intubated in 97.8 and 93.6% of studies. The duration of the study was significantly longer with the presence of a fellow trainee assisting in the procedure. Patient age and gender did not correlate with prep adequacy or cecal and ileal intubation rates, inadequate cleanout was significantly associated with impairment and incomplete studies.

**Conclusion:**

A one day, single agent, osmotic laxative (Polyethylene glycol) based cleanout regimen is effective in routine pre-procedure cleanout for standard colonoscopy in pediatric age range patients.

## Background

The adequacy of colon cleansing before colonoscopy is a principal determinant of the quality of colonoscopy in the pediatric as in the adult population. Of the several determinants of bowel prep including regimen, timing, diet, co-morbidities, and patient characteristics, preparation regimen is by far the most important predictor of successful procedure outcomes [1]. Among the many preparation regimens available, Poly-Ethylene-Glycol (PEG), an osmotic laxative, is the primary choice for bowel prep, with demonstrated safety and efficacy in children [2, 3] However, with several other regimens reporting comparable efficacy, no standard protocol exists for pediatric bowel prep.

A common alternative to PEG monotherapy for bowel prep is dual therapy with a stimulant (bisacodyl, senna) or combined stimulant–osmotic (Magnesium Citrate) laxative, which directly affects nerve, smooth muscle, and epithelial cells in the intestine to alter mucosal electrolyte transport [4]. A 2014 review by NASPHGHAN found that while PEG was the regimen of choice for monotherapy, dual therapy with PEG and stimulant laxative was used by between 36% (age 2–5 cohort) and 61% (age 12+ cohort) of physicians [5].

Stimulant laxatives, when used in the context of colonoscopy preparation are often associated with adverse outcomes including bloating, cramps and abdominal discomfort [4], and are even contraindicated in certain patient populations, including patients with renal disease, bowel obstruction, and those at risk for dehydration or electrolyte imbalance [6]. In the treatment of pediatric constipation, stimulant laxatives are recommended as a second-line therapy if a PEG-only regimen fails [7]. Despite these important caveats, stimulant laxatives remain a popular choice for dual-therapy bowel prep in pediatric colonoscopy.

The purpose of this study is to report the experience with a one day PEG-only bowel prep regimen in a pediatric cohort of patients studied at a tertiary pediatric gastroenterology service.

## Methods

Children’s Mercy Hospital in Kansas City MO, is tertiary, urban, standalone children’s hospital with dedicated inpatient and ambulatory gastroenterology services. Standard bowel prep protocol for all patients undergoing elective outpatient colonoscopy during the study period at this institution involved a one day age-based graduated dosing schedule of PEG3550 (Miralax©; 17 g/240 mL/dose) Patients were instructed to take 1 dose every hour for a minimum of 6 (< 4 years) – 10 doses, with additional doses as needed to return clear stool free of particulate matter. Dietary recommendations included clear liquids including electrolyte rich and non-red colored liquids through 2 - 4 hours pre-procedure all day or after breakfast (< 4 years age) the day before the scheduled procedure. All colonoscopies are tracked with an institutional ethical practice review approved survey instrument completed by the endoscopy staff, including providers immediately following the procedure. The survey includes patient demographics, prep quality, impairment, duration, and completion. A single abstractor reviewed data from all pediatric patients (age 0 – 20) who presented to our GI outpatient clinic for colonoscopy from September 2015 – September 2016 and were given standard PEG monotherapy. Cases in which > 1 of three primary outcomes measures (procedure duration, completion, impairment) were absent were excluded from the final sample.

Additionally, prospective data was collected on patients who presented to the GI outpatient clinic from October 2016 – January 2017 with otherwise identical inclusion and exclusion criteria using a slightly modified abstraction form. Prospective data included an additional field to record procedure deviations where secondary stimulant laxative was added. To characterize outcomes in PEG-only prep, deviations from prep protocol, including the use of additional laxatives and patient non-compliance, were excluded from the final analysis.

Outcomes of interest included quality of prep, procedure impairment, completion, and duration (defined as scope-in to scope-out). Quality of prep was categorized as; thin liquid, thick liquid, and solid matter. Impairment was recorded as a binary variable, based on physician assessment of bowel prep. Completion was measured as a binary variable for two levels – reaching cecum and terminal ileum when ordered. Additionally, retrospective data included a binary variable for the presence of GI Fellows. All binary variables were compared using Chi-Square Test. Procedure duration was compared between procedures performed by attending physicians and fellows using a Fischer Exact Test. Additionally, procedure duration was compared between first, second and third year fellows using a one-way ANOVA. *P*-value of 0.05 was used as a threshold of significance.

## Results

Retrospective data is summarized in Table 1. A total of 519 cases met all inclusion and exclusion criteria. Average age was 12.1 ± 4.2 years, with a male to female ratio of 0.64:1. Since cases were only excluded if > 1 primary outcome data field was missing, the total number of cases in each field represents those for which a response was submitted. There were no significant differences between age groups in terms of thin liquid (*p* = 0.241), thick liquid (*p* = 0.913), impairment (*p* = 0.783), reaching cecum (*p* = 0.485), or duration (*p* = 0.243). Significant differences were found in solids (*p* = 0.001) and reaching TI (*p* = 002).

**Table 1:** Quality of cleanout by age at the time of endoscopy: retrospective cohort

Age	Total	Sex (M)	Quality				Completion		Duration (Mean ± SD)
			Thin Liquid	Thick Liquid	Solid	Impairment	Cecum Reached	TI Reached	
1–5	47	21 (44.7%)	27 (57.4%)	5 (10.6%)	7 (14.9%)	6 (12.8%)	42 (89.4%)	33 (100.0%)	21.6 ± 10.2
6–9	96	38 (39.6%)	68 (70.8%)	14 (14.6%)	3 (3.1%)	8 (8.3%)	93 (96.9%)	85 (96.6%)	21.4 ± 10.3
10–13	136	61 (44.9%)	102 (75%)	20 (14.7%)	2 (1.5%)	13 (9.6%)	129 (94.9%)	123 (94.6%)	24.6 ± 12.1
14–16	172	51 (29.7%)	121 (70.3%)	26 (15.1%)	9 (5.2%)	22 (12.8%)	162 (94.2%)	157 (94.0%)	23.4 ± 11.8
17–20	68	31 (45.6%)	50 (73.5%)	8 (11.8%)	1 (1.5%)	8 (11.8%)	64 (94.1%)	57 (95.0%)	23.0 ± 10.7
p value		0.038	0.241	0.913	0.001	0.783	0.485	0.002	0.243
Measured	519	202	368	73	22	57	490	455	23.5 ± 11.9
Total	519	519	463	463	463	482	501	486	439
Percent	100.00%	38.90%	79.50%	15.80%	4.80%	11.80%	97.80%	93.60%	NA

Prospective data is summarized in Table 2. A total of 137 cases met inclusion criteria. 10 subjects were non-compliant with bowel prep regimen, while 4 other,required additional stimulant laxatives due to perceived poor prep. These cases were excluded from the final analysis. Table 3 summarizes the differences in procedure metrics based on prep quality, and found significant differences in procedure impairment (*p* < 0.001), reaching cecum (< 0.001), reaching TI (< 0.001), and procedure duration (0.011).

**Table 2:** Quality of cleanout by age at the time of endoscopy: prospective cohort

	Measured	Total	Percent
Total	123	123	100%
Demographics			
Age (Mean ± SD)	13.0 ± 3.7	123	NA
Gender (M)	60	123	48.78%
Outcomes			
Pre- procedure reported constipation	8	123	6.50%
Non-Compliant	10	[137]	7.30%
Additional Laxative	4	[127]	3.10%
Impairment	6	123	4.88%
Completion			
TI Reached	121	123	98.37%
Duration			
Duration/minutes (Mean ± SD)	27 ± 11.5	123	NA

**Table 3:** Impairment, completion of procedure and duration by quality of cleanout

Quality	Total	Impairment	Cecum	TI	Duration (Mean ± SD)
Thin Liquid	368	10/349 (2.9%)	352/355 (99.2%)	331/347 (95.4%)	22.2 ± 11.4
Thick Liquid	73	25/68 (36.8%)	70/71 (98.6%)	60/67 (89.6%)	26.4 ± 11.4
Solid	22	13/19 (68.4%)	15/21 (71.4%)	13/19 (68.4%)	24.8 ± 6.9
*P* value		< 0.001	< 0.001	< 0.001	0.011

A summary of Fellow procedure duration is provided in Table 4. Of a total of 439 recorded cases, 299 were performed by attending physician only, while 140 were performed by a GI fellow with an attending physician present. Table 3 also summarizes differences in procedure duration between first, second and third year trainee fellows. The *p* value for both comparisons was < 0.0001.

**Table 4:** Effect of fellow participation on procedure duration

	# of Cases	Duration/minutes (Mean ± SD)
Total	439	23.5 ± 11.9
Attending Only	299	22 ± 11.1
Fellow	140	27.1 ± 12.6
p value		< 0.0001
Fellow Yr1	69	30.2 ± 13.5
Fellow Yr2	64	21.8 ± 9.0
Fellow Yr3	22	31.0 ± 14.0
*p* value		< 0.0001

## Discussion

The purpose of this study was to characterize outcomes in a 1-Day PEG-only bowel prep in a pediatric population; pediatric gastroenterologists are generally split in their preference for PEG monotherapy or PEG-stimulant laxative dual therapy for colonoscopy preparation (5). The results of this study support the implementation of a one day PEG-only regimen with high adherence rates and good quality preparation and excellent colonoscopy completion rates.

The choice of a prep strategy in children has to balance between the conflicting desire toward a pristine cleanout with the disadvantages inherent to complex regimens including poor adherence, discomfort from dietary restrictions and poor adherence with complex instructions. This equation in turn needs to be refocused within the goals of colonoscopy in children which rarely, if ever, include detection of small lesions or polyps as in colon cancer surveillance and standard of care reliance on biopsy based diagnosis which decreases the onus for macroscopic assessment and high quality preparation.

Quality of prep with PEG-only prep was excellent. Solids found in colon during procedure is generally associated with poor prep, while the presence of only thin or thick liquids is considered adequate prep [8] . In our retrospective data, thin liquids were found in 79.48% of cases, thick liquids were found in 15.77% of all cases, and solids were found in only 4.75% of recorded cases, indicating overall adequate prep in > 95% of cases. Physician-determined impairment due to poor prep was noted in 11.8% of retrospective cases. Retrospective data included cases with patient deviation from protocol and additional stimulant laxative, and likely reflects expected prep impairment in a practice setting. In the prospective data, which included only protocol-adherent cases, prep-related impairment was only 4.88%, again indicating adequate prep in > 95% of cases. Only 4 cases (3.1%) required an alternative regimen. In all 4 cases, a parent directly contacted GI clinic staff after partial completion of the PEG regimen requesting additional laxative due to perceived ineffectiveness. In a similar comparison of regimens for constipation in children, 18% of patients given PEG monotherapy required additional laxatives [9].

Cleanout efficacy in our data is comparable to that of other studies of PEG-only regimen, which found cleanout efficacy rates as high as 89% and 95% [10, 11]. Notably, these other studies utilized a 4-day PEG prep of 1.5 g/Kg, a significantly longer and more intrusive protocol. Achieving similar cleanout rates with a lower-dose, one-day regimen is encouraging and significant. Additionally, patient adherence with regimen was also high; with 7.3% of patients being non-compliant with the assigned regimen. Decreased non-adherence rates compares favorably with other studies in which low volume PEG prep was used, in contrast to higher non-adherence found in studies using high volume PEG preparation, which ranged from 38 to 46% [12, 13].

Completion rates were also quite high, with cecum and terminal ileum visualization in 97.80% and 93.60%, respectively, in retrospective data, and terminal ileum visualization at 98.37% in the prospective group, comparable to results in similar studies using larger volume PEG prep solutions [12]. The results of this study are significant in showing comparable quality, impairment, compliance, and completion of prep in 1-day PEG bowel prep and high-volume, multi-day prep. A number of past studies have highlighted the contrast between superior efficacy of high-volume PEG solution and low patient tolerance and adherence to a multi-day, high-volume PEG prep [2, 12, 14]. Other groups have attempted to address this issue by exploring alternate delivery models such as split-dosing and low-volume PEG with added stimulant laxative [15–17].

Although the dominant alternative that has emerged to high-volume PEG monotherapy is low-dose PEG with stimulant laxative dual therapy, our observations suggest that high volume PEG limited to one day pre-procedure is effective and well tolerated. Further research is required to compare one day monotherapy with dual therapy in terms of safety, outcome based quality of prep, adherence and tolerability [5].

There are several limitations to this study. Our study focuses on an ambulatory care setting and our observations cannot be extrapolated to the inpatient setting. In the retrospective data set, deviations from the standard 1-day PEG regimen were not consistently recorded, including introduction of another cleansing agent or completion of the minimum 10-doses. Given the identical cleanout protocol,patient and provider population, we can extrapolate from the prospective cohort data that the likelihood of stimulant laxative administration, which is at least partially due to ineffective cleanout is approximately 3% and this should be factored with documented poor cleanout. Additionally, there were significant differences between retrospective and prospective data in terms of mean procedure time. We can partially attribute this discrepancy to differences in fellow participation in the two cohorts. Evaluation of impairment and poor prep due to cleanout protocol were ultimately subjective assessments made by the attending physician at the time of procedure. This may lead to significant variation in assessing impairment between providers, as well as overestimation of impairment due to providers attributing non-completion to prep quality rather than other factors such as technique. Another potential limitation is the lack of safety data in the cleanout forms. While this may be a significant exclusion, procedure nursing staff in our institution recall no incidences of medical or surgical adverse events related to cleanout protocol.

As an academic institution with an accredited pediatric fellowship program, we had the opportunity to analyze the impact of fellow participation on the duration of the procedure. Procedures performed by fellows were significantly longer (27.1 ± 12.6 min) than procedures performed by attending physicians only (22 ± 11.1 min), with a *p* < 0.0001. This discrepancy can be attributed to several factors including the normal progression in technical expertise, the impact of direct supervision and discussion during the case as well as the increased likelihood of fellows who are more advanced in their training to gravitate toward participating in more complex cases in order to meet their training requirements as well as out of interest. There was, in fact a significant difference in procedure duration between PGY 4 (30.2 ± 13.5 min), PGY 5 (21.8 ± 9.0 min), and PGY 6 (31.0 ± 14.0) fellows(*p* < 0.0001).

## Conclusion

1-Day PEG monotherapy appears to be an adequate bowel prep regimen for colonoscopy in the pediatric population. Based on the comparable levels prep quality, compliance, completion, of 1-Day prep with high-volume, multi-day PEG prep and other combined laxative solutions, we do not see the need for additional stimulant laxative to be added to the bowel prep regimen.

## Abbreviations

NASPGHAN: North American Society for Pediatric Gastroenterology Hepatology and Nutrition
PEG: Polyethylene Glycol
